# Automatic detection of health misinformation: a systematic review

**DOI:** 10.1007/s12652-023-04619-4

**Published:** 2023-05-27

**Authors:** Ipek Baris Schlicht, Eugenia Fernandez, Berta Chulvi, Paolo Rosso

**Affiliations:** 1 Universitat Politècnica de València, Valencia, Spain; 2 Independent Researcher, Valencia, Spain

**Keywords:** Health misinformation, Survey, Text mining, Machine learning

## Abstract

The spread of health misinformation has the potential to cause serious harm to public health, from leading to vaccine hesitancy to adoption of unproven disease treatments. In addition, it could have other effects on society such as an increase in hate speech towards ethnic groups or medical experts. To counteract the sheer amount of misinformation, there is a need to use automatic detection methods. In this paper we conduct a systematic review of the computer science literature exploring text mining techniques and machine learning methods to detect health misinformation. To organize the reviewed papers, we propose a taxonomy, examine publicly available datasets, and conduct a content-based analysis to investigate analogies and differences among Covid-19 datasets and datasets related to other health domains. Finally, we describe open challenges and conclude with future directions.

## Introduction

Internet is a popular and accessible source of health information (Percheski and Hargittai 2011; Marton and Choo 2012) that has even become a first choice for some individuals seeking information about their health conditions or medical advice before a consultation with a physician (Gualtieri 2009). Patients could be more engaged with their treatment decisions (Stevenson et al. 2007) and feel more confident as they acquire more information from the internet (Oh and Lee 2012). However, there is a significant amount of health misinformation on websites and social media (Waszak et al. 2018). Misinformation is disseminated through instant messaging apps and social media platforms and propagates faster and more broadly than legitimate news (Vosoughi et al. 2018).

The rapid spread of misinformation has led to new phenomena in information sharing like *infodemic*. The World Health Organization (WHO) 1 introduced the term *infodemic* following the onset of the Covid-19 pandemic. The term refers to a rapid increase in the spread of information, both false and true, about a specific topic such as a disease. The increase can be exponential and happen in a short time span caused by a particular incident like the Covid-19 pandemic. During this period of *infodemic*, people are bombarded by information and might find it difficult to assess which information is trustworthy and which one is not.

The spread of health misinformation has potential serious effects on society such as becoming a public health issue (Larson 2018). For example, the propagation of a hoax stating that *bleach-based alcohol kills the Covid-19 virus* caused hundreds of hospitalizations and even deaths in some countries (Islam et al. 2020b). Health misinformation can also evoke violence and hate towards certain groups, such as health workers (Huang and Liu 2020) or Asians as in the case of the Covid-19 pandemic (Wang et al. 2021; He et al. 2020).

Some websites (e.g. Quackwatch^2^) and fact/checking organizations (e.g. Snopes^3^) investigate the truthfulness of dubious health statements (aka fact/checking). However, fact/checking is a time-consuming process and requires health expertise. Automated fact/checking and misinformation detection have attracted many Computer Science (CS) researchers for some time.

There are several survey studies that examine general misinformation. Some of these surveys categorize misinformation based on theories from social sciences such as psychology (Shu et al. 2017; Zhou and Zafarani 2020). Others investigate only one type of misinformation like rumors  (Zubiaga et al. 2018). Only a few survey studies have focused on the health domain, such as Suarez-Lledó and Alvarez-Galvez (2021); Wang et al. (2019) who investigated health misinformation on social media platforms. However, these surveys reviewed papers from multidisciplinary literature, and only a few of the reviewed papers come from CS. Moreover, only a small number of studies evaluate datasets on health misinformation. Latif et al. (2020), D’Ulizia et al. (2021) are mostly concerned with Covid-19 datasets.

It is important to analyze the research specifically applied to health misinformation given that detection methods in other domains might not be easily applied to health misinformation. Characteristics, motivation, dissemination patterns, and receivers of misinformation could differ from one domain to another (Afsana et al. 2020). For example, in politics, crowd wisdom from social media has been widely used as a signal for identifying misinformation (Zubiaga et al. 2016). However, verifying health claims could require expert knowledge and crowd signals could be unreliable (Cui et al. 2020). Health/related claims, in turn, are commonly debunked by consulting scientific literature or the scientific community. To address this gap, in this paper we surveyed the CS literature in text mining and natural language processing to map the current approaches to detect health misinformation spread through search engines and social media. We also included datasets for evaluating detection methods that are not limited in scope to the Covid-19 pandemic. Furthermore, we included methods and datasets for languages other than English since misinformation is not limited to the English language and there is a need for assessing detection methods across multiple languages as evidenced by the current pandemic (Islam et al. 2020a).

In summary, our contributions in this survey are as follows:

- We conduct a systematic collection of studies focusing on automatic detection of health misinformation from the CS literature, including studies focusing on non-English corpora and not limited to Covid-19 like the other surveys. Therefore, our review is more comprehensive than other related surveys on health misinformation, with 43 papers from CS. Of the 43 papers, 19 papers present publicly available datasets for developing and evaluating machine learning methods.
- Since the methods and tasks for tackling health misinformation might differ according to aspects such as input type, health topic and misinformation type, we develop a taxonomy to categorize studies based on multiple criteria. Our taxonomy, therefore, covers more aspects than other health related surveys and focuses on CS perspective.
- In contrast to the other surveys, we conduct a content based analysis of the similarities and differences between the datasets related to the Covid-19 pandemic and those related to other health topics. We find that some linguistic and affective features of the two corpora are different.
- Lastly, we discuss the specific challenges of misinformation detection in the health domain and present future directions.

## Methodology

**Table 1:** The query used for the initial search

(health $\vee$ medical) $\wedge$ (disinformation $\vee$ misinformation $\vee$ conspiracy $\vee$ fake news $\vee$ mislead)$\wedge$ (machine learning $\vee$ text mining $\vee$ deep learning)	

Our research methodology was adopted from the guidelines of Kitchenham and Charters (2007) for conducting systematic reviews. The main steps of the guidelines include: select studies from databases and search engines, filter out papers based on a set of criteria, and lastly conduct a detail analysis of the final papers.

### Search keywords and data sources

(Swire-Thompson and Lazer 2019), (Sylvia et al. 2020) defined *health misinformation* as health/related claims that contradict current scientific knowledge. According to this definition, the veracity of a claim could change as the scientific community accepts new evidence. In this way, the problem of veracity when a piece of general information disseminates scientific results in the health domain presents particular challenges: the scientific community is used to the need to judge research results in the light of their limitations, but the general public has not this habit. As a result of this, exaggeration of findings is also a part of health misinformation. Addressing this particular problem, Sumner et al. (2014) argue that health news and academic press releases could misreport statements from a scientific publication by overemphasizing findings. In this case, even though the source of information is accurate, misrepresentation of research could have adverse effects, such as an increase in doubts in the effectiveness and safety of vaccines (Ramsay 2013).

In this paper we used both definitions of misinformation: health/related claims that contradict current scientific knowledge and exaggeration of findings that change the original value of the scientific research. From this perspective, we defined a search query to retrieve papers using machine/deep learning or text mining methods for analysis or detection of health misinformation. The query is shown in Table 1.

We used multiple sources to reduce biases and to have a more comprehensive search. The sources included ACL Anthology ,^4^ ACM Digital Library ,^5^ IEEE Xplore ,^6^ PubMed ,^7^ and AAAI .^8^ We used a library called findpapers 9 to search the sources except for ACL Anthology. In addition to these sources, we searched Papers with Code 10 for datasets and also investigated the references of the papers and of the surveys on health misinformation (Wang et al. 2019) and (Suarez-Lledó and Alvarez-Galvez 2021).

### Inclusion and exclusion criteria

Given the broad topic space of some of the sources used, the results from the query included some papers outside of the scope of the research interest of this survey. Moreover, some of the sources included papers that were not peer/reviewed. Therefore, to filter out results further, we defined an inclusion and exclusion criteria. The criteria defined is explained below:

- The papers must be written in English.
- The domain of the solution presented in the paper must be health misinformation.
- The methodology must be using machine/deep learning or text mining methods, and the details of the methodology and the related experiments must be given so that it is reproducible.
- The paper must be peer/reviewed, unless the paper is cited by multiple peer/reviewed papers. For instance,  (Cui and Lee 2020).

### Taxonomy of health misinformation

**Fig. 1:**
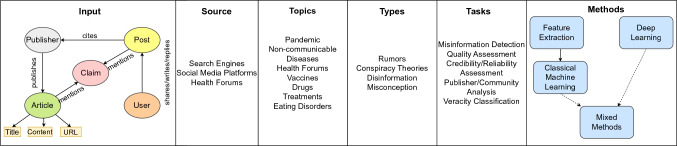
Taxonomy of Health Misinformation to organize the reviewed papers

We introduced a taxonomy of health misinformation to organize and annotate the survey results. Initially, the taxonomy had six dimensions guided by the research questions: inputs, source, topics, types, tasks, and methods. As we reviewed more papers, we updated the taxonomy to include new attributes. Figure 1 shows the final version of the taxonomy.

We first described inputs and sources of misinformation interaction on the taxonomy. As visualized in the taxonomy diagram, there is a claim that potentially contains misinformation. This claim is mentioned in articles of institutions, news media, which are indexed by search engines, and posts on social media or health forums. The posts can be written or shared by users, and they might also cite a link of an article discussing the same claim. The users might interact with each other by answering, quoting, or re/sharing the post.

To categorize the papers based on health topics, we leveraged the classification by Suarez-Lledó and Alvarez-Galvez (2021), which contains: vaccines, drugs, non/communicable diseases, pandemics, eating disorders, and treatments. In this classification, non/communicable diseases are chronic diseases that are not transmissible from one person to another, such as diabetes, or cancer. Pandemic is referred to an infectious disease spreading worldwide. Some of the papers we reviewed contain a mix of these topics, including drugs, treatments, and eating disorders, therefore, we annotated their topics as “various”.

As for misinformation types, we checked the main focus of each paper. We identified rumors, conspiracy theories, disinformation and misconception. We briefly explain their definitions according to Zannettou et al. (2019), Kapantai et al. (2021), Lewandowsky et al. (2012). Rumors are statements that are widely spread on social media; they are unverifiable at the time of the spread. Conspiracy theories are explanations of an event or situation resulting from a harmful plan organized by powerful entities. Disinformation is deliberately misleading and incorrect information. With this definition, fake news is part of disinformation (Kapantai et al. 2021). Therefore we relabelled the papers which examine fake news as disinformation. Misconception is information that is initially presented as true but later found to be false (Lewandowsky et al. 2012). The papers which used datasets including multiple types of misinformation were annotated as misinformation.

To identify tasks and methods, we examined the problem formulations and the methodology of the papers. As a result, we found misinformation detection, quality assessment, credibility or reliability assessment, publisher or community analysis, and veracity classification as the tasks tackled by the papers. We categorized the methods as classical machine learning and deep learning, as well as mix of both methods. The classical machine learning methods also include a step for feature extraction.

## Survey results

In this section, we present the results of the survey. We searched the data sources in April, 2022. Our query search resulted in 227 papers. After removing duplicate papers, filtering out papers based on our inclusion criteria, and adding relevant papers cited by these subset of papers, the final list of papers we reviewed was 43. Of the 43 papers, 56% of papers focused on misinformation during the pandemic. Other topics included non-communicable diseases, drugs, and vaccines. The majority of papers examined misinformation in social media (67%), while other platforms included news sites. Of all the papers reviewed, 72% studied an English/only corpus, and only 2 papers investigated multilingual or cross/lingual detection. Related to detection techniques, methods used deep learning (33%), traditional machine learning (28%), and a mix of both (37%). For experiments using deep learning techniques, pre/trained transformers (Vaswani et al. 2017) tended to outperform other models, whereas ensemble models like random forests outperformed most other traditional machine learning models. In the following subsections, we present the details of the papers based on the taxonomy.

### Topics, inputs, sources and types

**Table 2:** Topics, inputs, source of collection, language(s) that a paper focuses, and misinformation types that the reviewed papers tackle. Various topics contain one of topics such as drugs, non/communicable, etc. Interactions are engagements of source posts such as replies, retweets. FC: Fact checking, HNR: Health News Review. Misinformation refers to various types of misinformation such as conspiracies, rumors, and misleading claims, etc

Paper	Topics	Inputs	Source	Language(s)	Types
Mukherjee et al. (2014)	Drugs	Claim, Post, User	Health Forum	English	Misinformation
Ghenai and Mejova (2017)	Pandemic	Post	Twitter	English	Rumor
Kinsora et al. (2017)	Various	Post	Health Forum	English	Misinformation
Sicilia et al. ( 2018b)	Various	Post	Twitter	English	Rumor
Sicilia et al. (2018a)	Pandemic	Post	Twitter	English	Rumor
Ghenai and Mejova (2018)	Non/communicable	Post, User	Twitter	English	Rumor
Dhoju et al. (2019)	Various	Article	Facebook	English	Misinformation
Shah et al. (2019)	Vaccines	Article	Twitter	English	Misinformation
Kotonya and Toni (2020)	Various	Claim	FC Websites, HNR	English	Misinformation
Cui et al. (2020)	Non/communicable	Article	Websites	English	Misinformation
Afsana et al. (2021)	Pandemic	Article	HNR	English	Misinformation
Dai et al. (2020)	Various	Article, Post, User, Interactions	Twitter, HNR	English	Disinformation
Hossain et al. (2020)	Pandemic	Post	Twitter	English	Misconception
Li et al. (2020)	Pandemic	Claim, Article, Post, User, Interactions	Twitter	Multiple	Disinformation
Zhou et al. (2020a)	Pandemic	Article, Post, User	Websites and Twitter	English	Misinformation
Medina et al. (2020)	Pandemic	Post	Youtube	English	Conspiracy
Shahi and Nandini (2020)	Pandemic	Claim, Article	FC Websites	Multiple	Disinformation
Dharawat et al. (2020)	Pandemic	Claim, Post	Twitter	English	Misinformation
Kinkead et al. (2020)	Various	Article	Search Engine	English	Misinformation
Meppelink et al. (2021)	Vaccines	Article	Search Engine	Dutch	Misinformation
Wang et al. (2021b)	Vaccines	Post	Instagram	English	Misinformation
Panda and Levitan (2021)	Pandemic	Post	Twitter, Facebook	Multiple	Misinformation
Sarrouti et al. (2021)	Pandemic	Claim	Search Engine	English	Misinformation
Mattern et al. (2021)	Pandemic	Article, Post	Websites, Twitter	German	Disinformation
Alam et al. (2021)	Pandemic	Post	Twitter	Multiple	Disinformation
Haouari et al. (2021)	Pandemic	Claim, Post	Twitter	Arabic	Rumor
Zhao et al. (2021)	Non-communicable	Post, Interactions	Baidu	Chinese	Misinformation
Zuo et al. (2021)	Various	Article	HNR	English	Misinformation
Ayoub et al. (2021)	Pandemic	Claim, Post	Websites, Facebook, Twitter	English	Misinformation
Gerts et al. (2021)	Pandemic	Post	Twitter	English	Conspiracy
Argyris et al. (2021)	Vaccines	Post	Twitter	English	Conspiracy
Cerbin et al. (2021)	Pandemic	Post	Twitter	English	Misinformation
Jingcheng et al. (2021)	Vaccines	Post	Reddit	English	Misinformation
Smith et al. (2021)	Pandemic	Post	Twitter	English	Misinformation
Uyangodage et al. (2021)	Pandemic	Post	Twitter	Multiple	Misinformation
Upadhyay et al. (2021)	Various	Article	Search Engine	English	Misinformation
Du et al. (2021a)	Pandemic	Article	Search Engine	Chinese	Disinformation
Hayawi et al. (2022)	Pandemic	Post	Twitter	English	Conspiracy
Cui and Lee (2020)	Pandemic	Claim, Article, Post, Interaction	Websites, Twitter	English	Misinformation
Memon and Carley (2020)	Pandemic	Post, User	Twitter	English	Misinformation
Patwa et al. (2021)	Pandemic	Article, Post	FC Websites, Twitter, Facebook, Instagram	English	Disinformation
Yang et al. (2021)	Pandemic	Post, User	Weibo	Chinese	Disinformation
Di Sotto and Viviani (2022)	Various	Article, Post, Interactions	Twitter	English	Misinformation

In this section we present the health topics and misinformation types of the reviewed papers. We also review the inputs analyzed and their sources. Table 2 presents an overview of the results.

The most common health topics in the reviewed papers were about pandemics (58%). The most recent studies have focused on detecting Covid-19 misinformation, around 53% of papers evaluated models for Covid-19, however, some early studies (Ghenai and Mejova 2017; Sicilia et al. 2018a,  2018b explored detection of rumors related to the Zika virus on social media. Moreover, non/communicable diseases were also studied as a subject of health misinformation in 3 papers. In one of these, Ghenai and Mejova (2018) studied the characteristics of users and tweets of individuals sharing questionable information related to cancer treatments. Zhao et al. (2021) investigated automatic identification of misinformation about autism on Weibo, and Cui et al. (2020) proposed a model for detecting articles about cancer and diabetes. Other topics of health misinformation included drug treatments (Mukherjee et al. 2014) and vaccines (Shah et al. 2019; Meppelink et al. 2021; Argyris et al. 2021; Wang et al. 2021b). The papers that have topics annotated as "Various" in Table 2 contained a mix of topics on health misinformation such as drugs, treatments, and non/communicable diseases.

Among input types of the reviewed papers, some researchers focused on studying only articles (21%) and some only posts (51%). In terms of sources, the majority of studies analyzed social media platforms such as Twitter (53%), and to a lesser extent Facebook and Instagram.

Lastly, we annotated papers that focused on datasets including various type of misinformation. Sicilia et al. ( 2018b), Sicilia et al. (2018a), Ghenai and Mejova (2018), Ghenai and Mejova (2017), Haouari et al. (2021) investigated check/worthy rumors and doubtful statements. Other researchers investigated conspiracy theories about pandemics (Gerts et al. 2021; Medina et al. 2020) and vaccines (Argyris et al. 2021).

### Tasks and methods

**Table 3:** Tasks and best performing methods in each paper. $^1$ binary-class, $^2$ multi-class, $^3$ multi-label

Paper	Tasks	Best Method	
		Feature Category	Model
Mukherjee et al. (2014)	Credibility/Reliability Assessment$^1$	Linguistic, Affective, User	CRF
Ghenai and Mejova (2017)	Misinformation Detection$^1$	Linguistic, Sentiment, Platform, Medical, Miscellaneous	RDT
Kinsora et al. (2017)	Misinformation Detection$^1$	Linguistic, Affective, Medical, Network	RF
Sicilia et al. ( 2018b)	Misinformation Detection$^2$	Linguistic, Affective, User, Network	RF
Sicilia et al. (2018a)	Misinformation Detection$^2$	Affective, User, Network, Link	RF
Ghenai and Mejova (2018)	Community Analysis, Misinformation Detection $^1$	User, Sentiment, Linguistic, Readability, Medical, Timing	LR
Dhoju et al. (2019)	Publisher Analysis, Credibility/Reliability Assessment$^1$	Word Embeddings, Linguistic, Lexical, Link, Other	SVM
Shah et al. (2019)	Credibility Assessment$^3$	Tf/Idf	Ensemble (RF, SVM)
Kotonya and Toni (2020)	Veracity Classification$^2$	-	SciBERT, ExplainerFC
Cui et al. (2020)	Misinformation Detection$^1$	-	DETERRENT
Afsana et al. (2021)	Quality Assessment$^3$	Linguistic, Word Embeddings, Links, Other	SVM
Dai et al. (2020)	Misinformation Detection$^1$	-	SAF
Hossain et al. (2020)	Misinformation Detection$^2$	-	BERTScore, SBERT
Li et al. (2020)	Misinformation Detection$^1$	XLM-R Embeddings	dEFEND
Zhou et al. (2020a)	Credibility/Reliability Assessment$^1$	Word Embeddings, Image Features	Multimodal Network
Medina et al. (2020)	Misinformation Detection$^1$ (comments)	-	RoBERTa
	Misinformation Detection$^1$ (videos)	Comments, Conspiracy Percentage	SVM
Shahi and Nandini (2020)	Misinformation Detection$^1$	-	BERT
Dharawat et al. (2020)	Misinformation Detection$^2$	-	BERT with DA
	Misinformation Detection$^1$	-	dEFEND
Kinkead et al. (2020)	Quality Assessment$^3$	-	HEA-BERT
Meppelink et al. (2021)	Credibility/Reliability Assessment$^1$	CV	NB
Wang et al. (2021b)	Misinformation Detection$^1$	FastText (Hashtag, Text), VGG19 (Image)	Multimodal Network
Panda and Levitan (2021)	Misinformation Detection$^3$	-	mBERT
Sarrouti et al. (2021)	Veracity Classification$^2$	-	T5
Mattern et al. (2021)	Misinformation Detection$^1$	-	BERT with User Features
Alam et al. (2021)	Misinformation Detection$^3$	-	RoBERTa (English)
			XLM-R (Others)
Haouari et al. (2021)	Misinformation Detection$^1$	-	MARBERT
Zhao et al. (2021)	Misinformation Detection$^1$	Linguistic, Topic, Sentiment, Behavior	RF
Zuo et al. (2021)	Quality Assessment$^3$	Tf-Idf	GB
Ayoub et al. (2021)	Misinformation Detection$^1$	-	DistilBERT with SHAP
Gerts et al. (2021)	Misinformation Detection$^1$	N-grams	RF
Argyris et al. (2021)	Community Analysis, Misinformation Detection$^2$	CV	LR
Cerbin et al. (2021)	Misinformation Detection$^1$	Word Embeddings, Pyscho-Linguistic, Auxiliary, Social, Sentiment	GB
Jingcheng et al. (2021)	Misinformation Detection$^1$	Glove	CNN
Smith et al. (2021)	Misinformation Detection$^1$ $^2$	CV, Tf-Idf	Ensemble (NB, LR, SVM)
Uyangodage et al. (2021)	Misinformation Detection$^1$	-	mBERT
Upadhyay et al. (2021)	Misinformation Detection$^1$	DOM, Content, Link	Web2Vec
Du et al. (2021a)	Misinformation Detection$^1$	-	CrossFake
Hayawi et al. (2022)	Misinformation Detection$^1$	-	BERT
Cui and Lee (2020)	Misinformation Detection$^1$	-	dEFEND
Memon and Carley (2020)	Community Analysis	Socio-linguistics, Bot, Stance, Network	-
Patwa et al. (2021)	Misinformation Detection$^1$	Tf-Idf	SVM
Yang et al. (2021)	Misinformation Detection$^1$	-	Transformer
Di Sotto and Viviani (2022)	Misinformation Detection$^1$	Word Embeddings, Stylic, Emotion, Medical, Propagation, User	CNN with WE, Ensemble

In this section we present details about the tasks and methods of the reviewed papers. Table 3 presents an overview.

**Tasks** The majority of papers investigated methodologies or constructed datasets for misinformation detection. In general, this task is framed as a binary classification task. However, some papers investigated multi-class misinformation detection: e.g.  (Dharawat et al. 2020; Smith et al. 2021; Sicilia et al. 2018a,  2018b). Alam et al. (2021) annotated posts on Twitter according to multiple aspects such as check-worthiness, harmfulness to society, etc. for a fine/grained misinformation analysis. Other researchers tackled the misinformation detection task by reformulating the problem. For instance, Hossain et al. (2020) used retrieval and stance detection to identify whether a claim contained a known misconception, Argyris et al. (2021) and Medina et al. (2020) used stance detection respectively to group pro/anti-vaccination statements and conspiracy theories.

The task of quality assessment on articles and web pages has been examined by several papers. The task annotations were based on schemes developed by medical experts and journalists. The schemes evaluated medical research based on multiple aspects. DISCERN (Charnock et al. 1999), QIMR checklists and the criteria from the Health News Review (HNR)^11^ are examples of quality assessment criteria. Afsana et al. (2021) and Zuo et al. (2021) investigated machine learning models for automating the classification of criteria from HNR, while Kinkead et al. (2020) explored the algorithms for DISCERN criteria. Dai et al. (2020) unified the criteria of HNR into a binary classification for misinformation detection. Aside from investigating the quality assessment task, Shah et al. (2019) created new guidelines adapted from DISCERN and QIMR to train a model for evaluating credibility of vaccine-related web pages. Also, Dhoju et al. (2019) and Zhou et al. (2020b) employed machine learning for detecting credibility of publishers and Mukherjee et al. (2014) studied the credibility of user statements in a health forum related to drug side-effects.

Veracity detection, aka fact/checking task was applied at the claim-level in the papers reviewed. The aim of veracity detection is to verify a claim against a set of evidence retrieved from search engines. Sarrouti et al. (2021) and Kotonya and Toni (2020) explored veracity detection to verify health claims.

**Methods** The methods for detecting health misinformation range from standard feature-based machine learning to deep learning models, including transformers and explainable methods. Standard feature-based machine learning methods included Support Vector Machines (SVM), Random Forest (RF), Random Decision Tree (RDT), Naive Bayes (NB), Logistic Regression (LR), Gradient Boosting (GB) and XGBoost (Zhao et al. 2021; Sicilia et al. 2018a). Researchers using these models first extracted features to represent input data. Common features could be categorized into linguistic features, affective features Mukherjee et al. 2014; Dhoju et al. 2019; Afsana et al. 2021, and medical features such as medical reliability of URLs (Ghenai and Mejova 2018), or the number of biomedical terms (Di Sotto and Viviani 2022). Additionally, word embeddings extracted using Term Frequency-Inverse Document Frequency (TF-IDF), Count Vectorizers (CV) were commonly used as well.

On the other hand, some studies used deep learning methods such as Convolutional Neural Networks (CNN), bidirectional Gated Recurrent Units (biGRU) for detecting health misinformation or for the quality estimation task (Sicilia et al. 2018a; Zhou and Zafarani 2020; Cui et al. 2020; Dai et al. 2020). Wang et al. (2021b) and Zhou et al. (2020a) examined multimodal network classifiers taking text and images as inputs. Dai et al. (2020) performed the detection task with Social Article Fusion (SAF) (Shu et al. 2019) which combines news and social interactions with a network. One study (Upadhyay et al. 2021) trained a neural network whose input are content, Document Object Model (DOM) and URL features.

Recent studies evaluated transformers (Vaswani et al. 2017) applied to both social media content and news articles. Transformer/based architectures are pre-trained on very large text collections and subsequently their parameters are fine/tuned to specific tasks such as misinformation detection or quality estimation. As pre-trained models for the English datasets, BERT (Devlin et al. 2019) and RoBERTa (Liu et al. 2019) have been used by multiple studies. Also some researchers used domain specific transformers (Kotonya and Toni 2020) such as SciBERT (Beltagy et al. 2019) or applied a domain adaptation onto transformer embeddings (Dharawat et al. 2020) and (Hossain et al. 2020). Mattern et al. (2021) augmented BERT representation with the features representing users and post interactions. Hossain et al. (2020) used a sentence transformer (Reimers and Gurevych 2019) with semantic similarity between misconception and claim that was computed by BERTScore (Zhang et al. 2020) to identify misconceptions about Covid-19. As for non/English or multilingual datasets, XLM-R (Conneau et al. 2020) and multilingual BERT (mBERT) (Devlin et al. 2019) have been used by some studies. Additionally, Haouari et al. (2021) used MARBERT (Abdul-Mageed et al. 2021) to detect rumors in Arabic, and Du et al. (2021a) proposed a classifier that aggregates BERT embeddings of news sub-texts translated into English for detecting misinformation in Chinese. One study (Raffel et al. 2020) used text to text transformer (T5) for veracity detection.

Although deep learning methods have achieved state-of-the-art results, these approaches lack information on why they arrive at their predictions. Given the potential harm of health misinformation to society, transparency should be a key component of misinformation detection systems. A number of research studies investigated explainability of health misinformation detection. One research (Cui et al. 2020) proposed a model called DETERRENT that used knowledge graphs to explain why a news item had false claims. Another study (Kotonya and Toni 2020) used summarization methods and jointly trained the model with a fact/checking task. Furthermore, Ayoub et al. (2021) proposed using Shapley Additive exPlanations (SHAP) (Lundberg and Lee 2017) to explain the predictions of a DistilBERT model, a smaller version of BERT which was distilled by training a logistic regression model. Three studies (Li et al. 2020), (Dharawat et al. 2020) and (Cui and Lee 2020) used dEFEND which leverages co-attention network for highlighting important comments (Shu et al. 2019a).

Finally, some studies( Shah et al. (2019); Smith et al. (2021); Di Sotto and Viviani (2022)) used ensemble methods consisting of multiple machine learning algorithms to leverage strengths of different types of models.

**Discussion** Although transformers are the dominant method used in more recent papers, traditional machine learning models are still used for health misinformation detection due to their ease of implementation and because they provide a strong baseline to compare against more complex models. Traditional methods achieved competitive results on the quality estimation task, a multi-label task with news articles as inputs. Some limitations of transformers include the fact that they can only encode a limited number of tokens which may lead to ignoring parts of news articles important for detecting misinformation. On the other hand, transformers and other deep learning methods perform better at detecting misinformation when dealing with short, informal texts on social media. Majority of top performing models in recent papers were built using pre-trained transformers (e.g. BERT). Some points to keep in mind about the use of transformers include their ability to generalize and the computational power needed for training. In the event of a new epidemic, models trained with existing data might not generalize well when new medical terms and statements are introduced, requiring models to be fine/tuned or retrained. Transformers are expensive to fine/tune and require high computational power for fast inferences which might be particularly challenging for non/profit fact/checking organisations and newsrooms that have limited budgets. Moreover, because pre/trained transformers are trained on large amounts of training data, the fine/tuned models for health misinformation detection might inherit bias towards certain types of misinformation that could lead to incorrect classification and miss detecting harmful samples. An extensive evaluation should be encouraged to identify and mitigate these biases. We provide a discussion about other challenges and our recommendations in Sect. 5.

## Dataset evaluation

**Table 4:** Publicly available datasets of health misinformation. In Size: Small ($\le$5000), Medium ($\ge$5000 and $\le$10000), Large ($\ge$10000)

Topic	Dataset	Lang	Size	Date
Covid-19	Cui and Lee (2020)	en	Small	2019–2020
	Alam et al. (2021)	multi	Small	2020–2021
	Memon and Carley (2020)	en	Small	2019–2020
	Haouari et al. (2021)	ar	Medium	NA
	Patwa et al. (2021)	en	Large	NA
	Mattern et al. (2021)	de	Large	2020–2021
	Li et al. (2020)	multi	Large	NA
	Zhou et al. (2020a)	en	Small	2020
	Medina et al. (2020)	en	Small	2020
	Shahi and Nandini (2020)	multi	Medium	2020
	Hossain et al. (2020)	en	Medium	2020
	Dharawat et al. (2020)	en	Large	2019–2020
	Yang et al. (2021)	zh	Small	2019–2020
	Hayawi et al. (2022)	en	Large	2020–2021
Other	Kinsora et al. (2017)	en	Small	NA
	Dai et al. (2020)	en	Small	2009–2018
	Zuo et al. (2021)	en	Small	2006–2018
	Cui et al. (2020)	en	Medium	2014–2019
Mixed	Kotonya and Toni (2020)	en	Large	1995–2020

In this section we present an analysis of the datasets used in misinformation detection.

### Properties of the datasets

As seen in Table 4, the majority of datasets are related to Covid-19, only 4 papers tackle other health topics such as non/communicable diseases and only PubHealth (Kotonya and Toni 2020) contains both samples about Covid-19 and other health/related topics. Except for (Haouari et al. 2021), (Mattern et al. 2021) and (Zuo et al. 2021) whose samples are in Arabic, German and Chinese, respectively, all other datasets contain English samples. Some datasets (Shahi and Nandini 2020; Li et al. 2020; Alam et al. 2021) are in multiple languages covering more than 2 languages. Small datasets are the majority.

The sources of Covid-19 datasets generally come from fact/checking (FC) websites used to collect claims or articles. These datasets are later augmented to include related social media data such as posts, user-level data and propagation networks. Some datasets (e.g.  (Hayawi et al. 2022), (Alam et al. 2021) and (Memon and Carley 2020)), were collected directly from Twitter using topic-related keywords and were later annotated by experts. Hossain et al. (2020) and Dharawat et al. (2020) used the Covid-19 datasets of Dai et al. (2020); Zuo et al. (2021) used HNR and Cui and Lee (2020) used the Hoaxy API ,^12^ Snopes, a list of reliable sources to collect other health topics.

### Content-based analysis

**Fig. 2:**
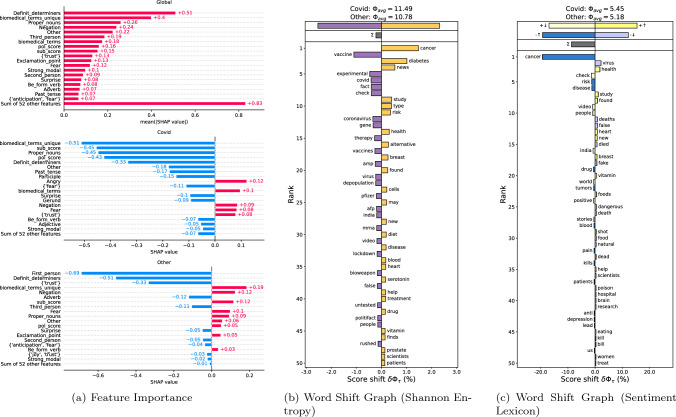
The results of content-based analysis on the Covid corpora and the corpora in other health topics. (**a**) The number of biomedical terms, some affective, and linguistic features distinguish the two corpora. (**b**) The left word shift graph shows the frequently used words for Covid and the right one shows the same for the other corpora. $\sum$ and $\phi$ scores indicate Covid-19 samples are more unpredictable than the other samples. (**c**) Negative words (-$\uparrow$) such as cancer, risk are used more often in the other corpora. The Covid corpora contains negative words (-$\downarrow$) related to mortality, factuality

To compare Covid-19 datasets with other health/related datasets, we conducted a content analysis on a subset of the datasets mentioned above. For the Covid-19 datasets, we selected the English datasets and excluded (Dharawat et al. 2020) since it was based on (Cui and Lee 2020). For the other group, we chose (Cui and Lee 2020), (Dai et al. 2020) and (Kinsora et al. 2017) and excluded (Zuo et al. 2021) since most of its samples are also contained in (Dai et al. 2020). We filtered out true samples and only used misinformation samples which resulted in 13094 samples for the Covid datasets, and 2193 for the other health/related dataset. Not all datasets had the same input types: some contained only articles or posts, while others contained multiple input types. We selected only titles from the datasets containing articles to unify the inputs into claims since titles mostly highlight claims. Also, we used source posts whenever a dataset had neither article nor claim.

**Importance of health features** After unifying the inputs into claims, we encoded the two corpora with health information features, including stylistic, emotional, and medical traits, using the implementation in  (Di Sotto and Viviani 2022). To see the features importance of the Covid-19 corpus and the other health/related corpus separately, we used an XGBoost classifier to classify a sample as either Covid or Other. We first split samples into a test set (20%) and a training set (80%). We then trained the classifier by applying 10-fold cross-validation on the training set. We used AUC as a metric to select the best classifier from the cross-validation. The best classifier received 0.883, indicating that these corpora are separable. Fig 2(a) presents a global summary of the feature importance of the two corpora as a cohort bar chart from SHAP (Strumbelj and Kononenko 2014). Some linguistic features such as the number of definite determinants, proper nouns, and negations were different between the two corpora. The words that describe the emotion of fear were the same in both corpora. However, surprise and trust-related words appeared more frequently in the Covid-19 corpus. Furthermore, the number of unique biomedical terms was an important feature for the other corpora as it covers more health topics.

**Word shift and sentiment analysis** To quantify the difference between the Covid-19 and the other health/related datasets, we first grouped all the pre-processed tokens into Covid and others. Then, we used a library called shifterator^13^ (Gallagher et al. 2021) to analyze word shifts among two corpora in terms of Shannon entropy and lexicon-based sentiment (Dodds et al. 2011). The results for both analysis are given respectively in Figs. 2(b) and 2(c). In Fig. 2(b), we can see that Covid-19 samples were more unpredictable than samples from the other corpus; this could be due to the nature of an infodemic. Besides the topical words such as Covid, we can see that vaccine, experimental, and fact were the top distinguished words on the Covid-19 corpus. Figure 2(c) visualizes the average sentiment of both corpora. The negative words were related to mortality of Covid-19, such as deaths, and dead, and factuality of the claims such as fake, false. The words like cancer, risk, and disease appeared less often in this corpus.

## Open challenges

This section presents the current issues on health misinformation and the potential solutions from an AI perspective.

**Data scarcity** Most misinformation datasets are about politics and most health misinformation datasets focus on Covid/19. Datasets in other domains of health misinformation, such as non/communicable diseases, should be constructed in order to prevent bias and implement more generalized models. There are not many studies exploring the feasibility and generalization of models trained on one health topic used for the detection of other health topics. This is particularly important to counteract misinformation during an infodemic.

Development of high-quality datasets for health misinformation is non-trivial. Ground truth labeling requires medical knowledge, and is thus costly and time-consuming. Therefore, the existing datasets usually lack enough samples to train deep learning methods. Transfer learning methods such as few-shot, zero-shot learning (Wang et al. 2019) are promising research directions to overcome this problem.

**Cross platform analysis** Most social media datasets in health misinformation are collected from Twitter. However, health misinformation is spread through instant messaging apps and other popular platforms such as Quora .^14^ The structure and user engagement of these apps and platforms and the propagation of misinformation are different from Twitter. Also, as popular platforms implement and improve existing policies against misinformation, misinformation actors can migrate to less regulated platforms (Nsoesie and Oladeji 2020). Developing cross-platform methods is crucial in order to transfer knowledge learned in one platform to another one.

**Multilingual datasets and methods** Social media platforms such as Twitter and Facebook have users from across the world. Trending misinformation in one country could be propagated to another country and become a new trend. To prevent this kind of issue, multilingual systems are required. These systems should be adaptive to different cultures and interests.

**Bridging science and public in explanations** Evidences for biomedical claims are often collected from scientific literature. Scientific articles contain domain specific knowledge which regular readers may find difficult to comprehend. The text simplification task (Ermakova et al. 2021) is a promising research direction for providing simplified explanations. Also, explanation systems should be evaluated by multiple stakeholders.

**Early detection of health misinformation** The consequences of health misinformation could be harmful for individuals or public health. Therefore, early detection of harmful information before it is disseminated is desired. User or publisher profiling could be a potential research direction.

## Conclusion

In this paper, we conducted a systematic review to identify methods and datasets for automatic detection and analysis of health misinformation. We also introduced a taxonomy to characterize and organize the reviewed papers. We noted that much attention has been paid to the development of approaches for combating Covid-19. There are also studies using state/of/the/art machine learning methods such as transformers. However, few studies have addressed other topics of health misinformation. Additionally, we presented a list of publicly available datasets in multiple languages from the articles reviewed. For comparison, we conducted a content-based analysis of Covid/19 and other health/related data/sets. We observed that their lexical and affective features differed. Finally, we identified open challenges in automatically detecting health/related misinformation and made recommendations for future research.

## Footnotes

1 https://bit.ly/3HIrqDG.

2 https://bit.ly/3nMVjuZ.

3 https://bit.ly/3NAvEjg.

4 https://bit.ly/3Nxrs46.

5 https://bit.ly/2KvJMOj.

6 https://bit.ly/2vbug3R.

7 https://bit.ly/3bG6ZfT.

8 https://bit.ly/3QYYjl6.

9 https://bit.ly/3Oz9cZr.

10 https://bit.ly/2BlKBn0.

11 https://bit.ly/3qkJwGl.

12 https://bit.ly/3OBqtB0.

13 https://bit.ly/3OEIVZB.

14 https://bit.ly/3QXOiEN.
